# Monitoring and Early Warning System: Regional Monitoring Strategy in Lombardy Region

**DOI:** 10.3390/epidemiologia6010007

**Published:** 2025-02-11

**Authors:** Sarah Cataldi, Elena Maria Ticozzi, Federica Morani, Annalisa Bodina, Maurizio Migliari, Gabriele Perotti, Massimo Lombardo, Fabrizio Ernesto Pregliasco, Danilo Cereda

**Affiliations:** 1 School of Medicine, Università Vita e Salute San Raffaele, 20132 Milan, Italy; 2 Department of Biomedical Sciences for Health, Università degli Studi di Milano, 20122 Milan, Italy; 3 Agenzia Regionale Emergenza e Urgenza, 20124 Milan, Italy; 4 Direzione Generale Welfare, UO Prevenzione, 20124 Milan, Italy

**Keywords:** surveillance, infectious diseases, public health, influenza

## Abstract

Background: This article examines the infectious disease surveillance system in the Lombardy region of Italy, with a focus on its response mechanisms to respiratory syndromes. This study aims to describe the alert system and the organizational procedures in place, assessing their effectiveness in managing health crises. Methods: This study is based on the analysis of Lombardy’s regional resolution No. 1125, developed by regional public health experts. Surveillance levels were categorized based on incidence thresholds and healthcare system impacts, establishing specific indicators and activation protocols. Information flows are managed through real-time data portals, enabling the real-time monitoring of COVID-19, influenza, and other infectious respiratory diseases. Results: A multi-level response system was established, with levels ranging from ordinary regimes to critical epidemic activation. Each level includes specific actions, such as resource reallocation, emergency department support, and the suspension of elective procedures. The use of technological tools, such as electronic health records, streamlined reporting processes, and real-time data flow management, has strengthened the region’s response capabilities. Conclusions: This study underscores the value of a structured, multi-level response system for infectious disease management, showing that a unified regional approach improves crisis response efficiency. It suggests that sharing activation indicators and protocols within the scientific community can help harmonize national and international responses to future pandemics. The system, while effective in its current context, may require adaptation for future health challenges.

## 1. Introduction

The decision-making process within the field of public health is multifaceted, influenced by an array of elements ranging from the accessibility of cutting-edge research evidence to local health concerns, the prevailing sociopolitical landscape, and the availability of public resources [1]. Of particular significance is the pivotal role played by population surveillance data, which serves as a cornerstone in providing decision-makers with crucial insights into pertinent health issues within the community context. This reservoir of data has played a vital role in influencing a wide range of policies, including regulations concerning the importation of animals, efforts targeting food recalls, and the promotion of public awareness campaigns aimed at tackling emerging environmental risks [2].

In the context of pandemic health emergencies, robust surveillance systems play a crucial role in re-establishing control [3,4]. These systems entail a spectrum of elements, including case definition, systematic data collection, the timely dissemination of findings, and the ongoing evaluation of the surveillance framework [5]. The monitoring and evaluation of the components of surveillance and response systems can be facilitated through the utilization of indicators that can be repeatedly measured over time. This approach can furnish valuable insights into the system’s status and identify areas necessitating improvement [6,7].

Since 1934, Italy has established a dedicated surveillance system for the notification of communicable diseases [8]. This system has undergone continuous refinement and enhancement, culminating in its computerization during the 1990s [9]. Since 2009, the system allows access to epidemiological data through weekly bulletins [10]. Furthermore, cooperative efforts, such as collaboration with the Lombardy Region during EXPO 2015, have contributed to the system’s further development [11]. Nowadays, the role of surveillance is so important that it has been systematized and developed as event-based surveillance (EBS), along with an expansion of the number of analysts through targeted training programs. In 2017, healthcare professionals from Regions, Maritime, Air, and Frontier Health Offices (USMAF)/Healthcare for Navigating Personnel (SASN) were involved in a national-scale training initiative to establish the first operational network of EBS analysts in Italy in 2018 [12,13].

Alongside common infectious disease notification in Italy, specialized surveillance systems have been implemented over time to gather timely and detailed information on specific diseases. Examples include surveillance systems for Legionellosis, Bacterial Meningitis, Tuberculosis (TB), Creutzfeldt–Jakob Disease, Hemophilus Influenzae, vector-borne diseases, measles, and rubella [9,14,15]. With the onset of the pandemic in 2020, integrated microbiological and epidemiological surveillance for COVID-19 began, which continuously and systematically collects, compares, and analyzes information on all confirmed cases of SARS-CoV-2 infection through molecular diagnosis in regional reference laboratories [16].

Consequently, the pandemic has instigated a re-evaluation of public health priorities, prompting a swift pivot towards preparedness and response strategies. These efforts encompass heightened attention to health professional training, meticulous contact tracing endeavors, and extensive mass vaccination campaigns [17,18,19]. Concurrently, the pandemic’s ripple effect has influenced disease surveillance programs unrelated to COVID-19, notably impacting cancer screening initiatives and necessitating adjustments in the management protocols for various health conditions, such as time-sensitive diseases [20]. The widespread integration of technological tools, notably smartphones and social media platforms, has significantly facilitated the acquisition of health surveillance data, thereby streamlining disease identification and monitoring efforts [21,22,23,24,25,26]. Therefore, this technological integration has catalyzed notable advancements in surveillance methodologies, marked by the implementation of innovative tools like electronic health records and wastewater surveillance systems [27,28].

During the COVID-19 pandemic, Lombardy emerged as the first Italian region significantly impacted by SARS-CoV-2 [29]. Consequently, the healthcare system in Lombardy underwent substantial changes, marked by an increased strain on emergency medical services (EMSs), overcrowding within emergency departments, and the imperative for novel organizational frameworks to confront post-pandemic hurdles [30,31,32].

The Lombardy region is the most populated Italian region, with 10 million residents. The healthcare system within Lombardy is managed by different agencies, for which their purpose is to organize health services for citizens in a harmonized manner. At the local level, the territorial and health surveillance systems are managed by the ATS (local health authorities), which are divided into eight distinct ATS across the region. Moreover, the entire regional emergency system is centrally administered by a sole agency known as AREU—Agenzia Regionale Emergenza Urgenza. Finally, the healthcare facilities responsible for delivering medical services are referred to as ASSTs (socio-sanitary territorial agencies), encompassing hospital facilities.

The purpose of this study is to describe the infectious disease surveillance system developed in the Lombardy Region, aiming to illustrate the alert and organizational mechanisms within the regional system.

## 2. Materials and Methods

The present article is based on the translation and analysis of Resolution No. 1125 of the Lombardy Region, developed by a regional expert working group [33]. The experts were public health professionals selected as representatives of the ATS, AREU, and ASST. They defined the tools and activities for surveillance with the aim of integrating the Regional Strategic–Operational Plan for Preparedness and Response to the Influenza Pandemic for the years 2023–2024.

The Lombardy Region currently operates several active surveillance systems aimed at monitoring respiratory syndromes. Specifically, during the COVID-19 pandemic, a dedicated system known as the “COVID surveillance system” was developed. This system serves to quantify various metrics, including total cases, intensive care admissions, swab testing, the Rt index, variant identification, the count of positive patients, hospitalizations, and fatalities related to COVID-19. It is closely linked to daily data flows recorded on the Cyber-Ark portal, facilitating the real-time monitoring of COVID-19 hospitalizations and intensive care unit admissions.

In contrast, data concerning the epidemiological and virological surveillance of influenza are collected and disseminated weekly via the “Influnet” portal. This is made possible through collaboration between the Virology Section of the University of Milan and the Italian National Institute of Health (ISS).

Regarding the laboratory surveillance of influenza-like illness (ILI) cases observed in emergency departments, selected clinical laboratories report their virological findings to the Lombardy Region through an established epidemiological reporting mechanism. This process involves submitting a file containing data collected throughout the week by noon on the Wednesday of the subsequent week utilizing a dedicated portal.

Furthermore, since October 2022, the Lombardy Region has implemented the SMI (Sorveglianza Malattie Infettive) system for reporting suspected and confirmed cases of infectious diseases. This system operates through the submission of reports by healthcare professionals, followed by validation through the procedures conducted by ATS subsequent to epidemiological investigations.

These systems encompass multiple levels of activation, each associated with specific criteria and indicators reflecting the incidence of respiratory syndromes, their impact on the healthcare system, and the required interventions.

## 3. Results

The working group utilized active surveillance systems within the region and established alert thresholds for each system. Furthermore, it delineated the response protocols of various regional system entities for each level of activation.

Subsequent paragraphs will delineate the different levels of activation, the adopted indicators and their threshold, and the healthcare system responses, highlighting the reorganization guidelines for the different regional structures (ATS, ASST, and AREU).

### 3.1. Activation Levels

The surveillance levels range from “ordinary regime”, characterized by low incidences and minimal impacts, to “epidemic activation”, indicating a critical impact on hospitals and a significant mobilization of resources to manage respiratory syndromes.

Table 1 outlines the different activation levels and their associated indicators.

For each activation level, specific actions are outlined, which must be implemented according to established criteria within the regional information system for pandemic monitoring, available to the local health authorities (ATS) and the socio-sanitary territorial agency (ASST). This will provide all stakeholders with a dedicated tool for verifying their ability to carry out the planned activities while also enabling the regional level to have an overall, cohesive, and structured overview.

#### 3.1.1. Ordinary Regime

**Figure 1:**
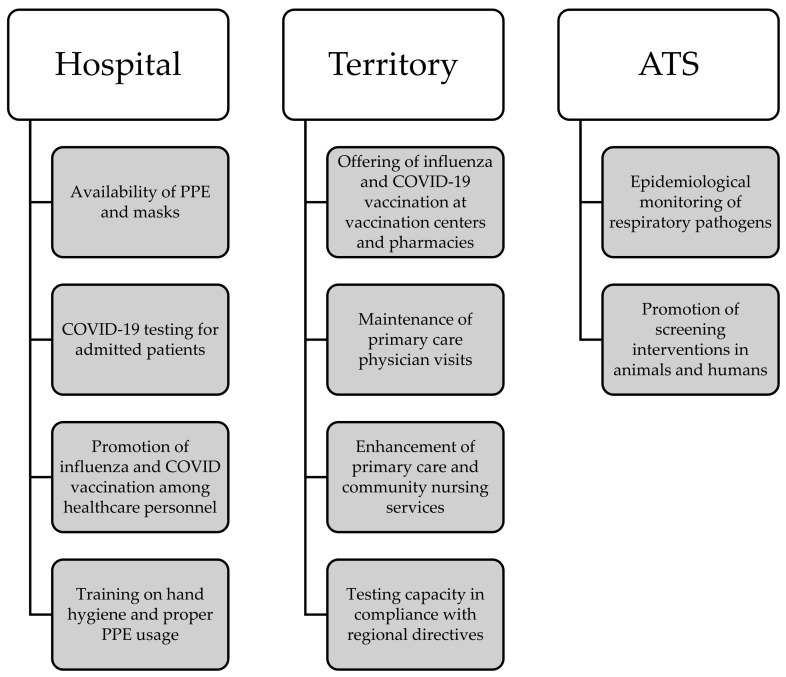
Visual overview of the main actions involved during the ordinary regime.

ILI incidence is <10/1000. The ordinary regime (Figure 1) encompasses a comprehensive set of preventive and management strategies deployed across hospital and territorial settings. Hospital-wise, paramount emphasis is placed on ensuring the ready availability of personal protective equipment (PPE) and masks, alongside the rigorous implementation of COVID-19 and other respiratory-pathogen-testing protocols for patients admitted to high-risk wards. Additionally, proactive promotion of influenza and COVID-19 vaccination among healthcare personnel is prioritized, with a targeted vaccination coverage surpassing 50%. These initiatives are further reinforced by structured training programs aimed at healthcare professionals to underscore the significance of hand hygiene practices and correct PPE utilization.

At the territorial level, efforts are concentrated on facilitating access to influenza and COVID-19 vaccination services via designated vaccination centers and community pharmacies. Concurrently, the continuity of primary care physician visits remains a focal point. Anticipatory planning involves bolstering primary care and community nursing services to meet potential surge demands, coupled with ensuring testing capabilities aligned with regional directives. Moreover, ATS leads interdisciplinary collaborations to meticulously monitor the epidemiological landscape of respiratory pathogens, including zoonotic transmission dynamics. Proactive advocacy for screening interventions targeting both animal and human cohorts further underscores the systemic approach.

#### 3.1.2. Territorial Activation

**Figure 2:**
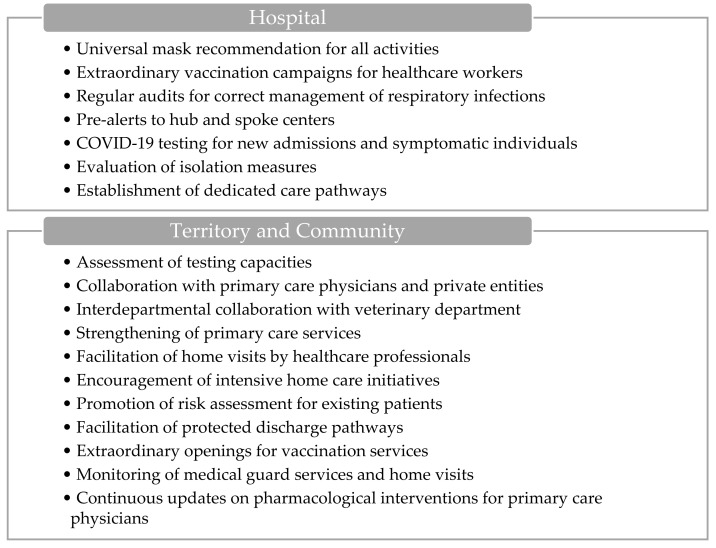
Visual overview of the main actions involved during territorial activation.

ILI incidence is >10/1000. The territorial activation level (Figure 2) introduces supplementary measures compared to the ordinary regime, aiming to enhance the response to the rising incidence of ILI. Within hospital settings, these measures encompass recommendations for universal mask usage, including outpatient care, alongside the implementation of extraordinary vaccination campaigns targeting healthcare workers. Additionally, regular audits are conducted to ensure the appropriate management of respiratory infections and isolation protocols across hospital departments. The hospital also initiates pre-alerts to hub and spoke centers, and COVID-19 testing is mandated for new admissions and symptomatic individuals, with periodic screenings for vulnerable patients. The evaluation of isolation strategies for symptomatic cases and the establishment of dedicated care pathways to ensure continuity of treatment are integral components.

On the community front, actions involve convening laboratory referents to assess testing capacities and potentially increase resources, including collaboration with primary care physicians and private entities. Interdepartmental collaboration with the veterinary department is fostered to monitor the epidemiological landscape of respiratory pathogens, emphasizing zoonotic transmission dynamics. Strengthening primary care services in critical areas and facilitating home visits by healthcare professionals, particularly physicians and community nurses, are key strategies. Moreover, intensive home care initiatives for frail individuals are encouraged to optimize care delivery.

Furthermore, ASSTs promote risk assessment for respiratory syndromes among existing patients and facilitate protected discharge pathways in partnership with local municipalities and other stakeholders involved in home care provision. Special arrangements are made for extraordinary openings to facilitate influenza and COVID-19 vaccination services, while the activation of medical guard services and the monitoring of initiated home visits are closely monitored. Finally, continuous updates regarding the use of antiviral treatments and other pharmacological interventions are disseminated to primary care physicians for informed decision-making and patient management.

#### 3.1.3. Emergency Department Activation

COVID-19 or flu ICU beds are <50.

**Figure 3:**
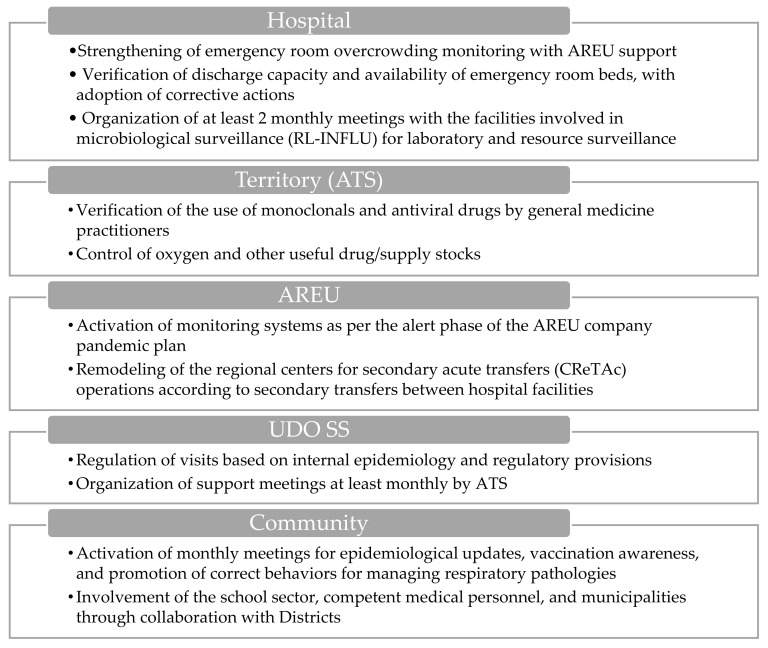
Visual overview of the main actions involved during the emergency department’s activation.

The activation of the emergency department (ED) (Figure 3) entails additional measures beyond standard protocols, including monitoring the time needed to transfer the patient from the ambulance stretcher to a bed in the emergency department.

At the territorial level, the ATS conducts assessments regarding the usage of monoclonal antibodies and antiviral medications by general practitioners, alongside evaluating the availability of oxygen and other essential medications and supplies. Additionally, AREU activates the monitoring systems outlined in the company’s pandemic plan and restructures the dispatch operational center as needed in order to facilitate secondary transfers between hospital facilities.

Within the hospital environment, there is a concerted effort to strengthen the monitoring of overcrowding indices in the ED, with support from AREU, and to evaluate discharge capacity and bed availability. The ATS conducts daily assessments of ED overcrowding indices and bed capacities for each facility, communicating this information to both the ATS and the Hospital Hub. Furthermore, there is a reinforcement of outpatient clinics for minor symptoms (white and green codes), and at least two monthly meetings are organized for levels I, II, and III of the microbiological laboratories involved in the surveillance system (RL-INFLU), as defined by DGR No. XII/63 of 27/03/2023, aimed at resource surveillance and laboratory delivery levels [34].

In socio-sanitary structures, such as territorial or domiciliary, diurnal, and residential structures (UDO SS), visits are regulated based on internal epidemiology and current regulatory provisions. ATS organizes support meetings at least monthly to facilitate coordination between the actors of the system.

Within the school community, ATS activates monthly meetings to provide epidemiological updates, promote vaccination, and remind stakeholders of appropriate behaviors for managing respiratory pathologies. These meetings involve various stakeholders, including health promotion departments, occupational medicine doctors, and municipalities through collaboration with the districts.

#### 3.1.4. Hospital Level 1 Activation

COVID-19 or flu ICU beds are >50. COVID-19 hospital beds are >500 (primary disease).

**Figure 4:**
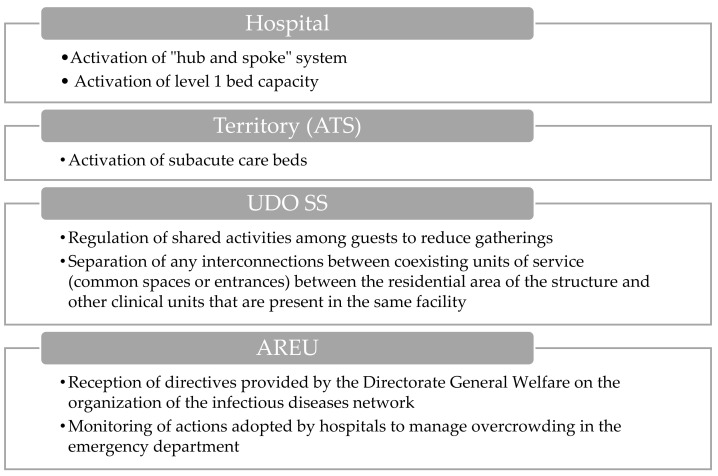
Visual overview of the main actions involved during the hospital level 1 activation.

During the activation phase of hospital level 1 (Figure 4), the implementation of the “hub and spoke” model in collaboration with AREU and territorial authorities assumes a crucial role. Simultaneously, the activation of subacute care beds in ASST in collaboration with ATS and territorial authorities is essential to facilitate hospital discharges and ensure improved continuity of care across the territory.

Moreover, the AREU oversees and monitors actions aimed at managing emergency department overcrowding, encompassing patient flow tracking, resource assessment, and intervention effectiveness evaluation to alleviate overcrowding.

Furthermore, the UDO SS facilities are tasked with regulating shared activities within healthcare facilities to mitigate overcrowding and minimize infection transmission risks. This involves implementing protocols and guidelines to organize communal activities in a manner that can guarantee social distancing effectively. Additionally, they must ensure the separation of interconnected units within the same facility, employing physical barriers or designated areas to prevent cross-contamination between different units.

#### 3.1.5. Hospital Level 2 Activation

COVID-19 or flu ICU beds are >100. COVID-19 hospital beds are >1000 (primary disease).

**Figure 5:**
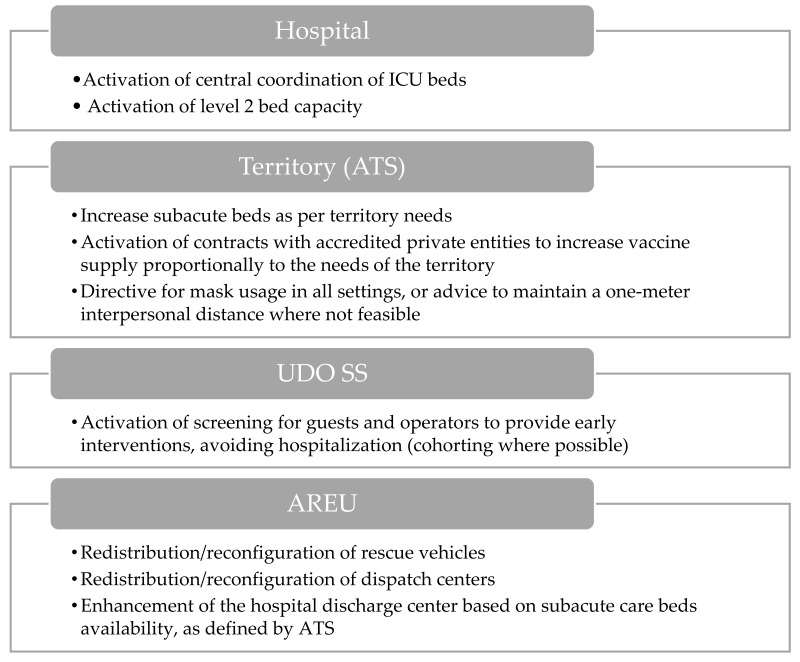
Visual overview of the main actions involved during hospital level 2 activation.

The level 2 hospital activation response (Figure 5) is distinguished by a supplementary escalation of interventions, in addition to those previously mentioned. Regarding hospitals, pivotal measures encompass increasing intensive care unit (ICU) beds and beds designated for COVID-19 patients, alongside the implementation of an additional tier of beds to accommodate heightened demands. Furthermore, the establishment of a centralized coordination mechanism is outlined to streamline the management of ICU beds. Concerning ATS, commitments include expanding the availability of subacute beds based on territorial needs. Additionally, contractual agreements with accredited private entities are proposed to ensure an equitable distribution of vaccines within the territory. Plans are also outlined for screening guests and staff to allow for early intervention and for advocating the use of masks in environments where social distancing is not possible.

The AREU is tasked with the redistribution and reconfiguration of rescue vehicles according to the requests of the regional health system. Furthermore, initiatives to reorganize operational centers are highlighted to enhance emergency management efficacy.

Lastly, emphasis is placed on strengthening the hospital discharge center to facilitate patient discharges, in alignment with the availability of subacute beds as defined by ATS.

#### 3.1.6. Hospital Level 3 Activation

COVID-19 or flu ICU beds are >150. COVID-19 hospital beds are >1500 (primary disease).

**Figure 6:**
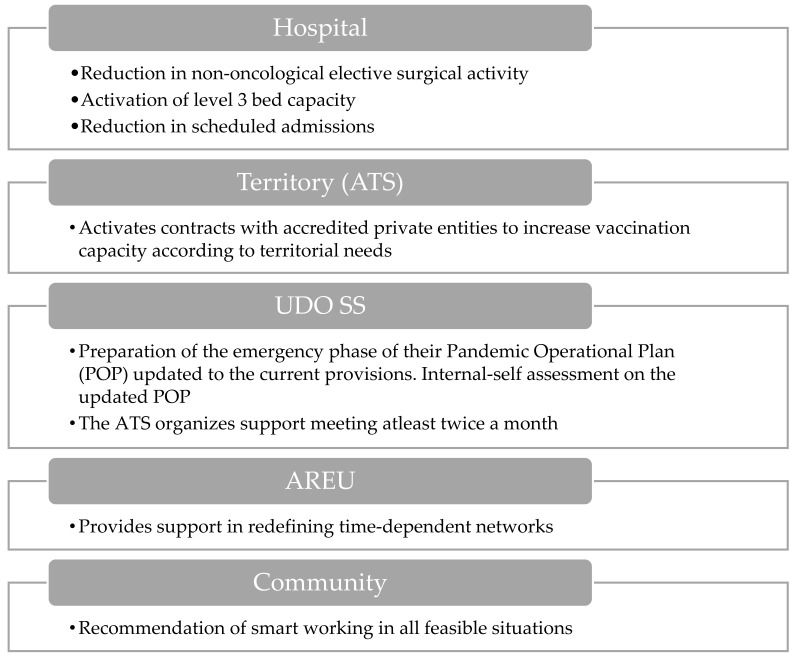
Visual overview of the main actions involved during the hospital level 3 activation.

During the hospital level 3 activation phase (Figure 6), it becomes imperative to significantly reduce elective surgical procedures and planned hospital admissions. A key role is assumed by ATS through the activation of contracts with accredited private groups to bolster vaccination capacities. Meanwhile, AREU provides crucial support in redefining time-dependent pathologies networks.

Within the healthcare system, the structures of UDO SS engage in territorial interventions and coordination efforts. Furthermore, there is a focus on the preparation and revision of Pandemic Operational Plans (POPs) to align with current guidelines, along with the internal self-assessment of these plans. Additionally, recommendations are made for the implementation of smart working practices where feasible in order to mitigate the risk of pathogen transmission.

#### 3.1.7. Epidemic Activation

COVID-19 or flu ICU beds are >300. COVID-19 hospital beds are >3000 (primary disease).

**Figure 7:**
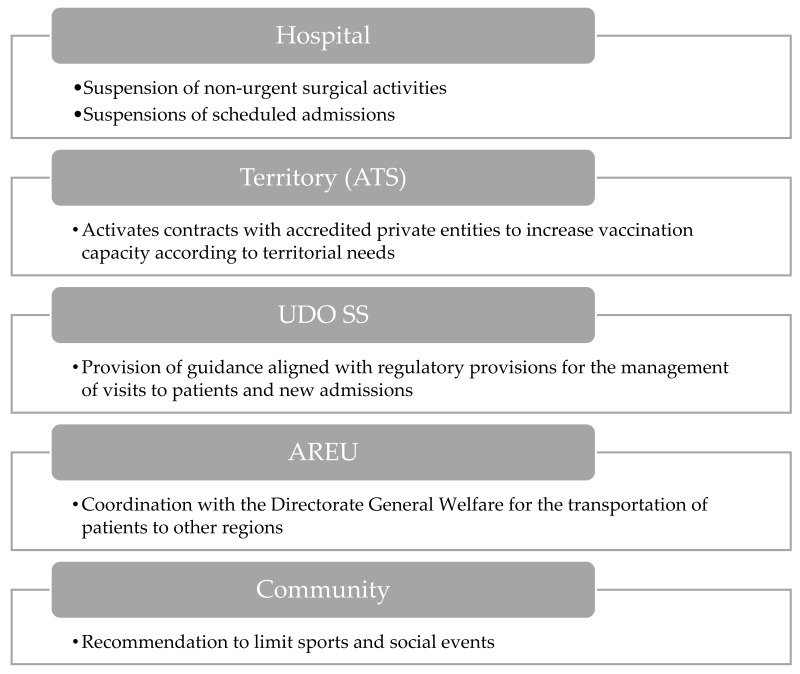
Visual overview of the main actions involved during epidemic activation.

The final escalation in response to a respiratory virus epidemic is the activation of the epidemic phase (Figure 7). In the hospital context, the suspension of non-urgent surgical procedures and planned admissions reallocates resources to accommodate the rising influx of COVID-19 patients.

At the territorial level, collaboration between ATS and accredited private groups aims to enhance vaccination efforts, ensuring alignment with regional needs. Additionally, AREU’s coordination facilitates patient transfers to other regions, easing the burden on local healthcare systems.

Within the structural part of the UDO SS, guidance ensures adherence to regulatory protocols for patient management, minimizing transmission risks during visits and admissions.

Recommendations for the community and school environments include limiting sports and social events to reduce opportunities for viral transmission in public gatherings.

#### 3.1.8. Hospital Bed Capacity

The expansion of hospital bed capacity emerges as a pivotal strategy in the effective management of the COVID-19 pandemic and the protection of public health. In Lombardy, healthcare institutions (hub and spoke centers) have categorized bed capacity according to different levels of care: level 1, level 2, and level 3.

At level 1, hospitals provide basic medical care for COVID-19 patients, encompassing 702 hub beds and 220 spoke beds across all institutions, serving as the frontline defense against the virus, and catering to patients with mild to moderate symptoms.

At level 2, hospitals have a total capacity of 984 hub beds and 330 spoke beds, managing patients with more severe symptoms who require specialized medical attention and interventions.

Level 3 represents the highest tier of medical response to a possible pandemic. With a combined capacity of 1276 hub beds and 440 spoke beds, level 3 facilities are equipped to provide intensive care for critically ill patients. These beds are equipped with advanced life support systems, including ventilators and extracorporeal membrane oxygenation (ECMO) machines, to support patients with severe respiratory distress and organ failure.

### 3.2. Adopted Measures

The presented model led to the adoption of measures listed in Table 2.

## 4. Discussion

This article, to the best of the authors’ knowledge, stands as one of the few that highlights an organizational aspect of surveillance. While numerous articles focus on predictive mathematical models, our emphasis lies on the challenges and opportunities provided by organizational models. Indeed, understanding how data flow can provide vital information to organizational decision-making is immensely beneficial [35,36].

It is crucial to underscore that while the organizational plan outlined in our research represents the most suitable approach at present, it may not be universally applicable in the context of future pandemics. Economic resources, staffing availability, and the evolving nature of pandemics must all be considered. Indeed, our pandemic plan involves transitions between different levels that necessitate actions and entail complex activities; however, the rapid spread of pathogens can possibly hinder the ability of some of the involved actors to execute the defined steps [37].

It is noteworthy to emphasize that the decisions formulated at this stage are influenced by the prevailing attitudes and operational practices among health workers (e.g., handwashing, mask use), as these factors have been demonstrated to impact pathogen spread [38,39,40]. Attitudes within the general population can also significantly influence pathogen transmission, underscoring the necessity for periodic updates to pandemic guidelines and plans and encompassing vaccination attitudes, cross-border mobility, and the use of personal protective equipment in public settings. Therefore, in order to be able to implement public health projects and plans, it is also beneficial to adopt educational projects aimed at the general public [41].

Despite these considerations, the definition of a comprehensive pandemic plan proves to be a valuable and essential element in ensuring that structures are aware of the necessary actions and activities to be undertaken by all pertinent health entities when addressing this scenario.

The dissemination and sharing of a pandemic plan and activation indicators within the scientific community serve as crucial elements for understanding how the operational part of public health mobilizes and utilizes warning systems. Furthermore, such sharing can aid in defining a practical model for national or international use, thereby standardizing health responses to potential pandemics.

## 5. Conclusions

Our study outlines the infectious disease surveillance system in the Lombardy Region, highlighting alert thresholds and organizational mechanisms. Based on Resolution No. 1125 of the region, our article stands as one of the few to emphasize the organizational aspect of surveillance. While the outlined plan suits the current context, it may not be universal for future pandemics, considering available resources and the evolving nature of diseases. Sharing the plan and activation indicators within the scientific community can contribute to standardizing pandemic responses nationally or internationally.

## Figures and Tables

**Table 1:** Levels of activation and surveillance indicators for respiratory syndromes in Lombardy during health emergencies.

Activation Level	Description	Indicator
Ordinary Regime	Low incidence of respiratory syndromes No significant impact on hospital management or intensive care	ILI incidence < 10/1000 inhabitants
Territorial Activation	Moderate incidence of respiratory syndromes No significant impact on hospital management or intensive care	ILI incidence > 10/1000 inhabitants
Emergency departments activation	Increased emergency department visits Presence of difficulties in managing respiratory syndromes COVID-19 or influenza has no significant impact on hospital wards	Emergency Department Overcrowding Time: 75th percentile value of overcrowding time exceeding 60 min in more than 3 adjacent Emergency Departments for more than 3 days. Overflow Indicator: Exceeding the 91st percentile of the number of patients received daily and historically in more than 3 adjacent emergency departments for more than 3 days. COVID-19 or flu ICU beds < 50
Hospital level 1 activation	COVID-19 or influenza begins to impact hospital management Overall hospital system functionality preserved	COVID-19 or flu ICU beds > 50 COVID-19 hospital beds > 500 (primary disease)
Hospital level 2 activation	Significant impact on hospital management due to COVID-19 or influenza Reorganization of hospital resources Overall hospital system functionality preserved	COVID-19 or flu ICU beds > 100 COVID-19 hospital beds > 1000 (primary disease)
Hospital level 3 activation	COVID-19 or influenza significantly impact hospital management. Reduction in regular hospital activities	COVID-19 or flu ICU beds > 150 COVID-19 hospital beds > 1500 (primary disease)
Epidemic activation	Critical impact on hospital management due to COVID-19 or influenza. Suspension of regular hospital activities	COVID-19 or flu ICU beds > 300 COVID-19 hospital beds > 3000 (primary disease)

ILI: influenza-like syndrome; ICU: intensive care unit.

**Table 2:** Implemented measures in Regione Lombardia.

Date	Action	Flu Incidence	COVID-19 Incidence	COVID-19 Cases in Intensive Care Unit	COVID-19 Cases Hospitalized	Severe Flu Cases	Cumulative Severe Flu Cases
23 November 2023	Preparation for Territorial activation	8.8/1000	110/100.000	10	484	6	6
29 November 2023	Beginning of Territorial Activation	14.8/1000	140/100.000	26	559	0	6
6 December 2023	Operative indications for an effective management of the Territorial Activation	14.3/1000	129/100.000	39	650	8	14
15 December 2023	Hospital level 1 activation	17.2/1000	117/100.000	49	1109	27	41
16 December 2023	Hospital level 2 activation	17.2/1000	129/100.000	51	1136	1	42
18 December 2023	Hospital level 2 activation: expansion of the infectious disease surveillance system	20/1000	123/100.000	50	1133	9	51
19 December 2023	Hospital level 2 activation: creation of an alert unit, with the objective of monitoring the emergency department system’s overcrowding	20/1000	112/100.000	50	1167	7	58
21 December 2023	Hospital level 2 activation: activation of additional hospital beds	20/1000	104/100.000	46	1141	10	68
6 February 2024	Return to ordinary regime	8.1/1000	7/100.000	16	342	5	174

## Data Availability

No new data were created or analyzed in this study. Data sharing is not applicable to this article.
